# Cannabinoids Inhibit Acid-Sensing Ion Channel Currents in Rat Dorsal Root Ganglion Neurons

**DOI:** 10.1371/journal.pone.0045531

**Published:** 2012-09-19

**Authors:** Yu-Qiang Liu, Fang Qiu, Chun-Yu Qiu, Qi Cai, Pengcheng Zou, Heming Wu, Wang-Ping Hu

**Affiliations:** 1 Department of Pharmacology, Hubei University of Science and Technology, Xianning, Hubei, P R China; 2 Hubei Furen Pharmaceutical Corporation Ltd, Shinanqiao, Tongcheng, Hubei, P R China; Dalhousie University, Canada

## Abstract

Local acidosis has been found in various pain-generating conditions such as inflammation and tissue injury. Cannabinoids exert a powerful inhibitory control over pain initiation via peripheral cognate receptors. However, the peripheral molecular targets responsible for the antinociceptive effects of cannabinoids are still poorly understood. Here, we have found that WIN55,212-2, a cannabinoid receptor agonist, inhibits the activity of native acid-sensing ion channels (ASICs) in rat dorsal root ganglion (DRG) neurons. WIN55,212-2 dose-dependently inhibited proton-gated currents mediated by ASICs. WIN55,212-2 shifted the proton concentration–response curve downwards, with an decrease of 48.6±3.7% in the maximum current response but with no significant change in the EC_50_ value. The inhibition of proton-gated current induced by WIN55,212-2 was almost completely blocked by the selective CB1 receptor antagonist AM 281, but not by the CB2 receptor antagonist AM630. Pretreatment of forskolin, an AC activator, and the addition of cAMP also reversed the inhibition of WIN55,212-2. Moreover, WIN55,212-2 altered acid-evoked excitability of rat DRG neurons and decreased the number of action potentials induced by acid stimuli. Finally, WIN55,212-2 attenuated nociceptive responses to injection of acetic acid in rats. These results suggest that WIN55,212-2 inhibits the activity of ASICs via CB1 receptor and cAMP dependent pathway in rat primary sensory neurons. Thus, cannabinoids can exert their analgesic action by interaction with ASICs in the primary afferent neurons, which was novel analgesic mechanism of cannabinoids.

## Introduction

Tissue acidosis is a common factor found in various pain-generating conditions such as inflammation, ischemia, infection, tissue injury and tumor development [1], [2]. The local drop in pH is detected by peripheral nociceptor and plays an important role in the pathological pain [3], [4]. It is well known that tissue acidosis produces pain. For instance, direct application of an acidic solution into the skin induces non-adapting pain [5], [6]. Acid-sensing ion channels (ASICs) are proton-gated cation channels and mediate the acid-evoked currents. To date, seven subunits of ASICs (1a, 1b1, 1b2, 2a, 2b, 3, and 4) encoded by four genes have been identified [7]. All other ASICs, except ASIC4, are present in primary sensory neurons including dorsal root ganglia [8], [9]. The activation of ASICs is likely to play a role in the perception of pain in these conditions associated with tissue acidosis [10]. Increasing evidences suggest that ASICs are involved in inflammatory and neuropathic pain [11], [12], [13]. ASICs inhibitors have been shown to relieve pain in a variety of pain syndromes [14], [15]. Thus, ASICs appear as a potential therapeutic target for pain therapy.

Cannabinoids have been used for thousands of years to provide relief from suffering. Cannabinoids modulate nociceptive processing via their cognate receptors, cannabinoid receptor 1 and 2 (CB1 and CB2). CB1 receptors are constitutionally active and abundantly expressed in the nociceptive primary sensory neurons [16], [17], [18]. CB2 receptors, on the other hand, are expressed in a variety of immune cells and microglia. There are considerable evidences supporting a role for cannabinoids in the modulation of pain. Cannabinoids are found to inhibit pain responses to noxious thermal and mechanical stimuli, as well as nociceptive behaviours in the formalin test [19], [20], [21]. In models of chronic inflammatory pain and neuropathic pain, cannabinoid ligands have been shown to reduce thermal and mechanical hyperalgesia and attenuate the pain behaviour [22], [23], [24]. Thus, cannabinoids are effective as analgesics in acute pain as well as chronic pain [25].

Genetic deletion of CB1 receptors further confirmed their role in cannabinoid-induced analgesia [26], [27]. Agarwal et al. [28], by using specific deletion of CB1 receptors in nociceptive neurones of primary sensory ganglia, concluded that the contribution of CB1 receptors expressed on the peripheral, rather than the central, terminals of nociceptors is paramount to cannabinoid-induced analgesia. The antihyperalgesic effect of cannabinoids is inhibited by the CB1 receptor antagonist [23]. Site-specific administration of agonists and antagonists suggests that CB1 receptors inhibit pain responses by acting at peripheral sites [29], [30]. However, the molecular targets responsible for the antinociceptive effects of peripherally applied cannabinoids are still poorly understood. In this study, we show that WIN55,212-2, a cannabinoid receptor agonist, inhibited the activity of native ASICs in the sensory neurons isolated from rat dorsal root ganglia (DRG).

## Results

### Proton-gated Currents in Rat DRG Neurons

**Figure 1:**
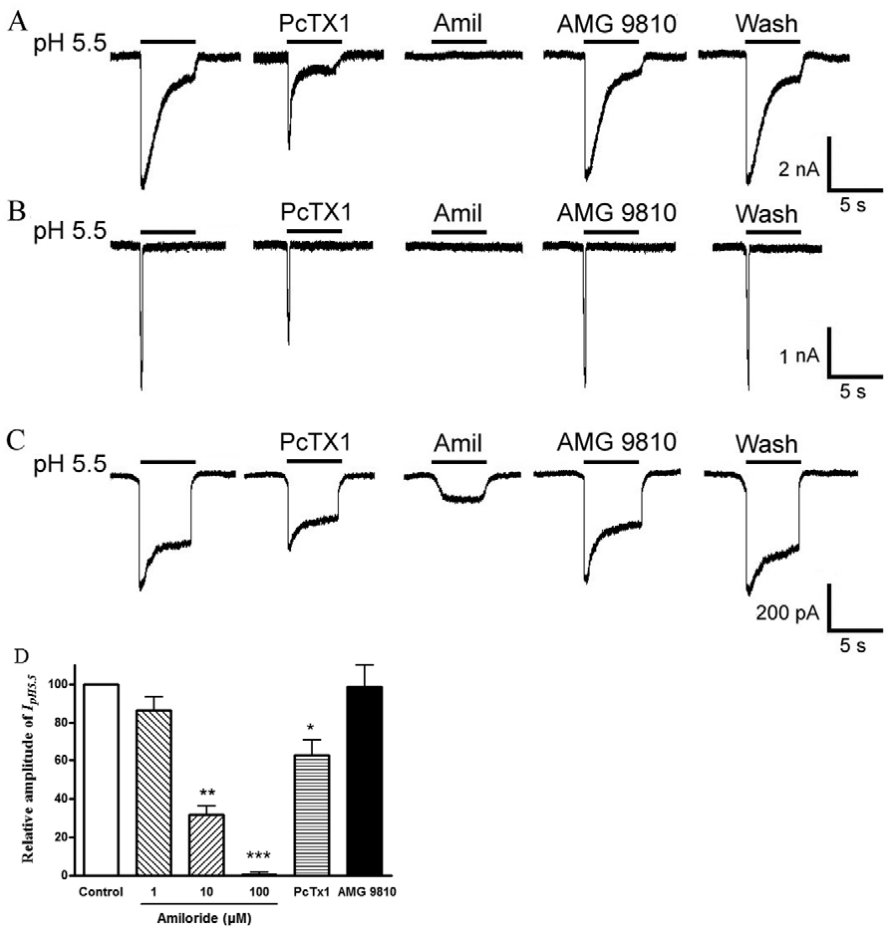
Three types of proton-gated currents in rat DRG neurons. Three types of proton-gated currents were recorded in DRG neurons. Example traces of a slow-inactivating current followed by a small sustained component (A), and a fast-inactivating current (B), a sustained current with a small transient phase (C). All three types of proton-induced currents could be partly inhibited by 10 nM PcTx1, a specific antagonist of homomeric ASIC1a channels. These currents could be completely or partly blocked by 100 µM amiloride (Amil), a broad-spectrum ASIC channel blocker. The TRPV1 blocker AMG 9810 (1 µM) has little effect on the transient peak phases of three types of acid currents. The bar graph in (D) shows relative amplitude of peak currents induced by pH 5.5 after application of amiloride, PcTx1 and AMG 9810. Proton-induced currents were evoked by extracellular application of a pH 5.5 solution for 5 s. *P<0.05,**P<0.01, ***P<0.001, one way analysis of variance followed by post hoc Bonferroni’s test, compared with control. Each column represents the mean ± SEM of 8–11 neurons.

Freshly isolated neurons from rat DRGs in the range of 15–35 µm were used in the present study. In most native DRG neurons (76.9%, 87/113), an inward current (I_pH5.5_) was evoked by application of a pH 5.5 solution for 5 s in the whole-cell patch-clamp configuration. Under our experimental conditions, three main types of acid-induced currents were observed on the basis of their amplitudes and inactivation time courses. A slow-inactivating transient inward current followed by a small sustained component was present in 56.3% (49/87) of neurons (Fig. 1A). A fast-inactivating current was present in 9.2% (8/87) of cells (Fig. 1B). And a sustained current with a small transient phase was present in 34.5% (30/87) of neurons (Fig. 1C). The maximum amplitude of the slow- and rapid-inactivating transient current was at least 3 times greater than that of the sustained current. The transient currents could be clearly divided into two groups on the basis of their inactivation time constants. The slow-inactivating transient current had a mean inactivation time constant of 1921.1±226.4 ms, whereas the rapid-inactivating current had a shorter inactivation time constant (436.5±62.9 ms, P<0.001). We established objective criteria for the distinction of these current types. Time constants of inactivation <800 ms were classified as rapid-inactivating current, while those >800 ms were classified as slow-inactivating current.

All three types of acid-induced currents could be partly inhibited by 10 nM PcTx1, a specific antagonist of homomeric ASIC1a channels (Fig. 1). An ASIC3-like current was revealed after the inhibition of the slow-inactivating transient current by application of PcTx1 (Fig. 1A, the second current trace). Relative amplitude of peak currents induced by pH 5.5 was 62.9±7.8% of control after application of PcTx1 in eleven DRG neurons tested (Fig. 1D, P<0.05). The slow- and rapid-inactivating transient currents could be completely blocked by 100 µM amiloride, a broad-spectrum ASIC channel blocker (Fig. 1A and B). Blockade of the acid-evoked currents by amiloride was dose-dependent. After the application of 1, 10 and 100 µM amiloride, relative amplitude of peak currents were 86.4±7.1%, 31.7±4.7% and 0.4±1.5%, respectively (Fig. 1D, n = 8). In addition, amiloride (100 µM) also could completely block the peak phase of the sustained current. However, it only could partly block the sustained phase of the sustained current, suggesting other channels such as TRP channels may be involved (Fig. 1C). We further measured proton-gated currents in the presence of AMG 9810 (1 µM) to block proton activation of TRPV1 [31]. Similarly, three types of acid-induced currents were also observed after application of AMG 9810. The transient peak current amplitudes of three types of acid currents (pH5.5) did not significantly change in the presence of AMG 9810, and relative amplitude of peak currents induced by pH 5.5 was 98.6±11.3% of control (Fig. 1D, n = 8, P>0.1). However, the sustained phase of the sustained current could be partly inhibited by AMG 9810 (Fig. 1C). Thus, the slow- and rapid-inactivating transient currents may be ASIC currents, and the sustained currents may be mediate by ASICs and TRPV1. To functionally characterize ASIC currents, we mainly observed the slow- and rapid-inactivating transient currents in this study.

### Effect of WIN55,212-2 on Proton-gated Currents

**Figure 2:**
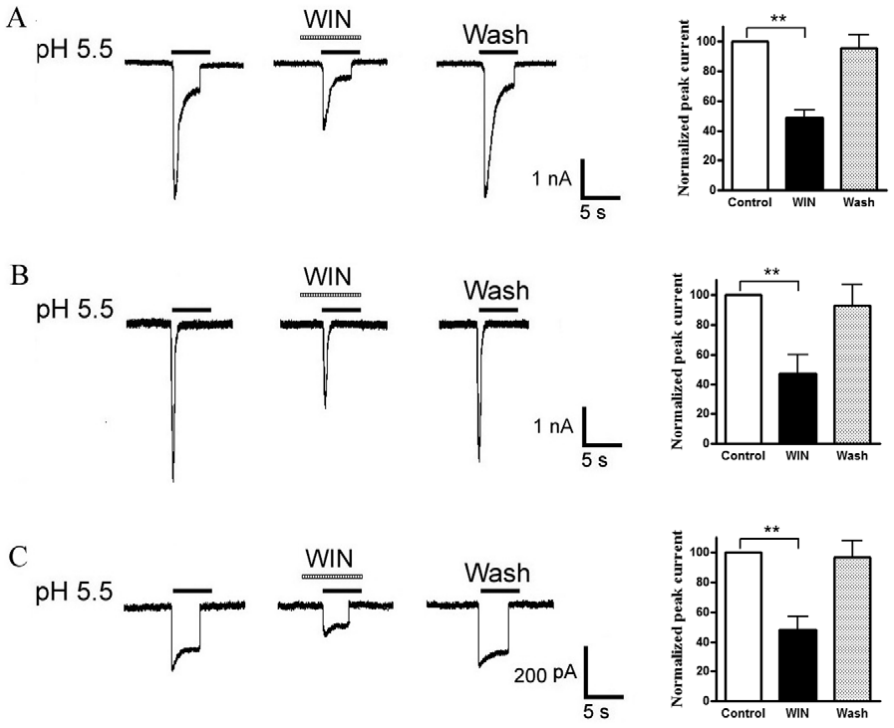
Inhibition of the three types of proton-gated currents by WIN55,212-2. Pre-application of WIN55,212-2 (WIN, 10^−7^ M) for 60-s decreased the peak phases of all three types of proton-induced currents (Original current traces in left). Bar graphs in right panel show currents normalized to control (100%, white column). Data in all bar graphs are shown relative to control. Error bars show ± SEM. Statistical tests were performed on raw data using pairing *t*-test, and significance is shown as follows: *P<0.05, **P<0.01. n = 6/each column.

In the majority of the neurons sensitive to acid stimuli (74.7%, 65/87), we observed that all three types of proton-evoked currents were inhibited by the pre-application of 10^−7^ M WIN55,212-2, a CB receptor agonist (Fig. 2). The amplitude of peak phase of slow-inactivating current decreased to 48.8±5.0% of that before application of WIN55,212-2 (n = 6, P<0.01), the fast-inactivating current to 46.8±12.9% (n = 6, P<0.01), sustained current to 48.4±8.6% (n = 6, P<0.01), respectively (Fig. 2 bar graphs in right panel). Thus, WIN55,212-2 showed similar modification on all three types of currents. To address the stereospecificity of the WIN55,212-2 for modulating acid currents, we also tested effect of WIN55,212-3, a cannabinoid-inactive enantiomer of WIN55,212-2, on proton-evoked currents. Unlike inhibiting effect of WIN55,212-2, the amplitude of acid currents was 98.4±8.2% of the control with pretreatment of WIN55,212-3 (n = 6, P>0. 1, paired *t*-test), suggesting no effect WIN55, 212-3 on proton-evoked currents.

**Figure 3:**
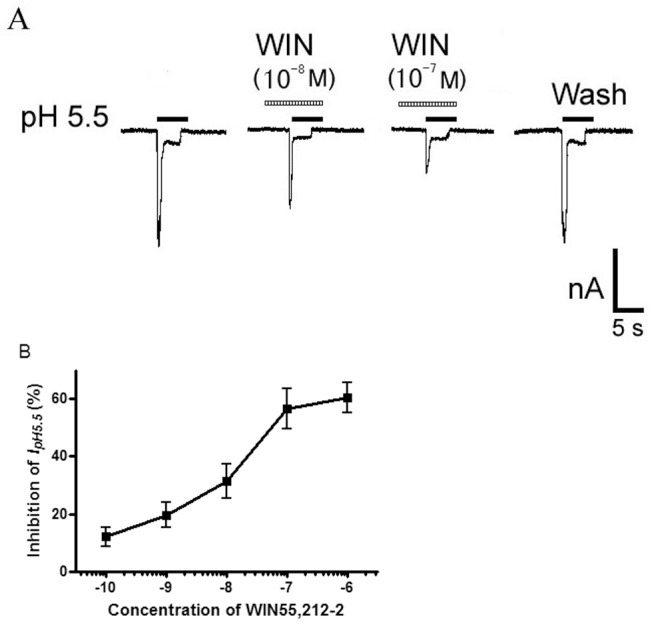
Concentration-dependent inhibition of proton-gated currents by WIN55,212-2. A. Sequential current traces illustrate the inhibition of proton-induced currents by different concentration of WIN55,212-2 (WIN) on a DRG neuron with membrane potential clamped at −60 mV. Proton-gated currents were elicited by application of pH 5.5 for 5-s durations. The pretreatment time for WIN55,212-2 was 60 s. B. WIN55,212-2 decreased proton-gated currents (I_pH5.5_) in a concentration-dependent manner (10^−10^–10^−6^ M). Each point represents the mean ± SEM of 7–10 neurons.

We next investigated whether the inhibition of proton-gated currents was dependent on the concentrations of WIN55,212-2. Fig. 3A shows that the amplitudes of I_pH5.5_ further decreased when the concentration of WIN55,212-2 increased from 10^−8^ M to 10^−7^ M. Figure 3B shows the dose-response curve for WIN55,212-2 in the inhibition of proton-gated currents. Each dose was examined in 7–10 neurons. The amplitude of I_pH5.5_ decreased stepwise with an increase of WIN55,212-2 from 10^−10^ M to 10^−6^ M. The WIN55,212-2 caused the maximum effect (60.5±5.4%, n = 8) at concentration of 10^−6^ M. The results indicated that WIN55,212-2 inhibited the proton-gated current in a dose-dependent manner.

### Concentration-response Relationship for Proton-gated Currents with and without Pretreatment of WIN55,212-2

**Figure 4:**
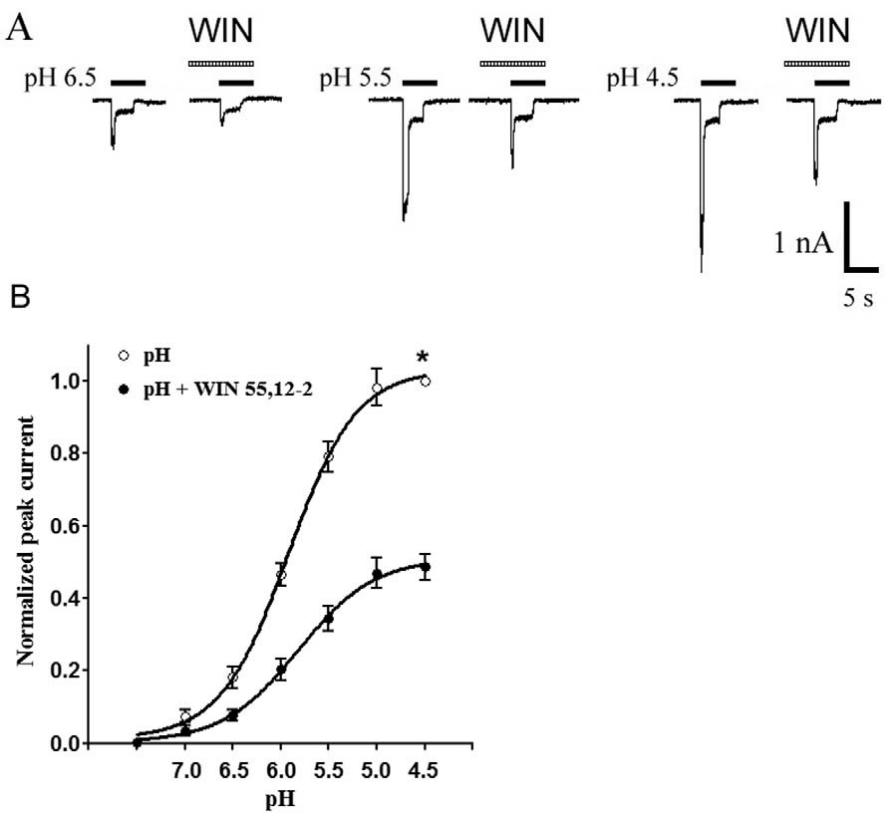
Concentration-response relationship for proton with or without the pre-application of WIN55,212-2. A. Sequential currents evoked by different pH in the absence of WIN55,212-2 (WIN) and presence of WIN. B. The concentration-response curves for proton with or without WIN55,212-2 (10^−7^ M) pre-application. The concentration-response curve for proton with WIN55,212-2 pretreatment shifted downwards. Each point represents the mean ± SEM of 7–11 neurons. All current values were normalized to the current response induced by pH 4.5 applied alone (marked with asterisk).

Figure 4 shows the concentration-response curves for proton in the absence and presence of WIN55,212-2 (10^−7^ M). Seven to ten neurons were detected for each point of curves. It can be seen that (i) the concentration- response curve for proton with pretreatment of WIN55,212-2 was shifted downwards as compared with the control; (ii) the EC_50_ value in both curves was no statistical difference (pH 5.82±0.11 with WIN55,212-2 pretreatment vs pH 5.92±0.06 without WIN55,212-2 pretreatment; P>0.1, Bonferroni’s *post hoc* test); (iii) the maximal amplitude of proton-gated currents at pH 4.5 after pretreatment with WIN55,212-2 decreased to 48.6±3.7% as compared with the control (n = 8, P<0.01, Bonferroni’s *post hoc* test); and (iv) the threshold pH values of both the curves were basically the same.

### The Receptor and Intracellular Signal Transduction Mechanisms Underling Suppression of Proton-gated Currents by WIN55,212-2

**Figure 5:**
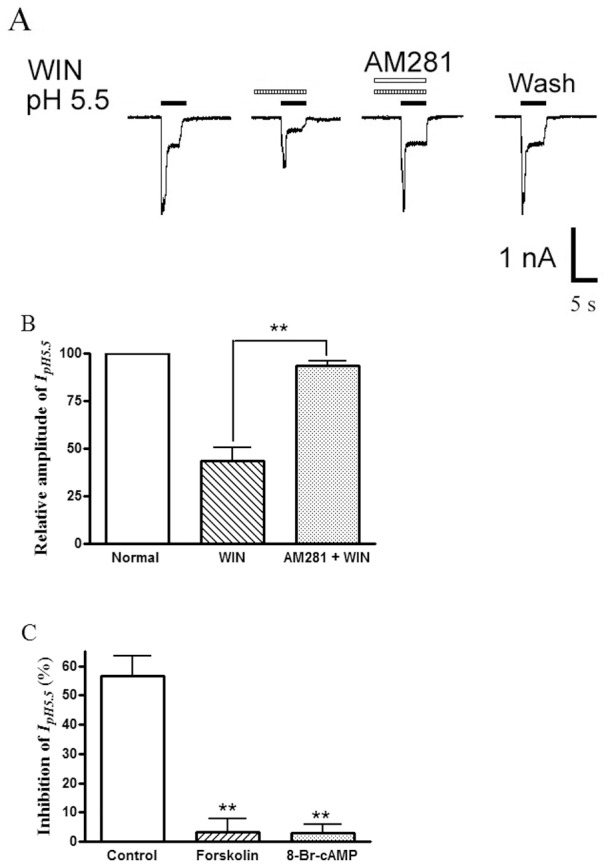
The receptor and intracellular signal transduction mechanisms underling inhibition of proton-gated currents by WIN55,212-2. The current traces in (A) and the bar graph in (B) show that the inhibition of I_pH5.5_ by WIN55,212-2 (WIN, 10^−7^ M) pre-applied alone was abolished by the co-application of WIN55,212-2 and AM281 (10^−6^ M), a selective CB1 receptor antagonist **P<0.01, one way analysis of variance followed by *post hoc* Bonferroni’s test, n = 8. The bar graph in (C) shows the percentage decreases in the I_pH5.5_ induced by WIN55,212-2 (10^−7^ M) in control and pre-treatment of forskolin and 8-Br-cAMP conditions. The inhibition of WIN55,212-2 on proton-gated current was blocked by application of forskolin (10^−5^ M) or 8-Br-cAMP(10^−3^ M). **P<0.01, one way analysis of variance followed by *post hoc* Bonferroni’s test, compared with control, n = 7/each column.

To verify whether the suppression of proton-gated currents by WIN55,212-2 was mediated by the CB receptor, we examined the effect of AM 281, a selective CB1 receptor antagonist, on the WIN 55,212-2-induced inhibition of proton-gated currents. As shown in Figure 5A and B, the suppression of I_pH5.5_ by pretreatment with WIN 55, 212-2 (10^−7^ M) was almost completely reversed by the addition of 10^−6^ M AM 281 (n = 8, P<0.01,one way analysis of variance followed by *post hoc* Bonferroni’s test). However, the suppression of WIN 55, 212-2 was not effected by the addition of the CB2 receptor antagonist AM630. WIN 55, 212-2 (10^−7^ M) had a 57.5±7.0% inhibiting effect on proton-gated currents, while WIN 55,212-2 caused also a 54.1±7.9% inhibition in the presence of the AM630 (10^−6^ M) (n = 8, P>0.01, unpaired t-test).

CB1 receptors belong to Gi/o protein-coupled receptor family, and the activation of the receptors leads to a cascade of events that inhibit adenylyl cyclase (AC) and therefore decrease the intracellular cAMP level [32]. To further explore intracellular signal transduction mechanisms underling suppression of proton-gated currents by WIN55,212-2, an experiment using forskolin (an AC activator) and 8-Br-cAMP was carried out. As shown in Figure 5C, the inhibition of WIN55,212-2 on proton-gated current was completely blocked by application of forskolin (10^−5^ M) or 8-Br-cAMP(10^−3^ M). Suppression of I_pH5.5_ induced by WIN55,212-2 was 56.5±7.0% (n = 10) in the control experiment. In contrast, the suppression of I_pH5.5_ induced by WIN55,212-2 was 3.3±4.7% after treatment of forskolin (n = 7, P<0.05, post hoc Bonferroni’s test), and 2.8±3.0% after treatment of 8-Br-cAMP (n = 7, P<0.01, post hoc Bonferroni’s test).

Taken together, these results suggest that WIN 55,212-2-induced inhibition of proton-gated currents is mediated through the CB1 receptor and cAMP signaling pathway.

### Effect of WIN55,212-2 on Proton-evoked Action Potentials of Rat DRG neurons

**Figure 6:**
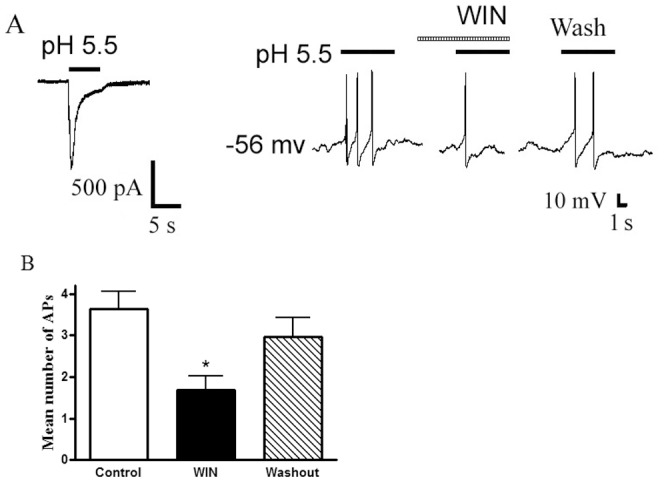
Effect of WIN55,212-2 on proton-evoked action potentials of rat DRG neurons. A. Original current and action potentials recordings from the same DRG neuron. Left panel, pH 5.5 induced an inward current with voltage-clamp recording. Right panel, pH 5.5 produced action potentials with current-clamp recording in the same neuron. The pretreatment of WIN55,212-2 (WIN, 10^−7^ M) decreased the acid-induced the number of action potentials. B. Bar graph shows the effect of WIN55,212-2 on the number of action potentials produced by pH 5.5 acid perfusions. After 30 min washout of WIN55,212-2, acid-evoked action potentials recovered to control condition. *P<0.05, paired t-test, compared with control, n = 8/each column.

Under current-clamp conditions, an acid stimulus (pH 5.5, 5 s) could trigger bursts of action potentials in rat DRG neuron, whereas it also induced an inward current with voltage-clamp recording in the same cell (Fig. 6A). The pre-application of WIN55,212-2 (10^−7^ M, 60 s) produced an inhibition of the acid-induced action potentials. The mean number of action potentials was 3.6±0.4 during exposure to pH 5.5 for 5 s in eight neurons tested. In contrast, the mean number of action potentials decreased to 1.7±0.3 after the pre-treatment of WIN55,212-2 (n = 8, P<0.05, paired t-test) (Fig. 6B). After 30 min washout of WIN55,212-2, the mean number of action potentials evoked by acid were 2.9±0.5, which was not significance difference with control condition (3.4±0.4, n = 8, P>0.1, paired t-test) (Fig. 6B). These results indicated that WIN55,212-2 inhibited proton-induced excitability of rat DRG neurons.

### Effect of WIN55,212-2 on Nociceptive Responses to Injection of Acetic Acid in Rats

**Figure 7:**
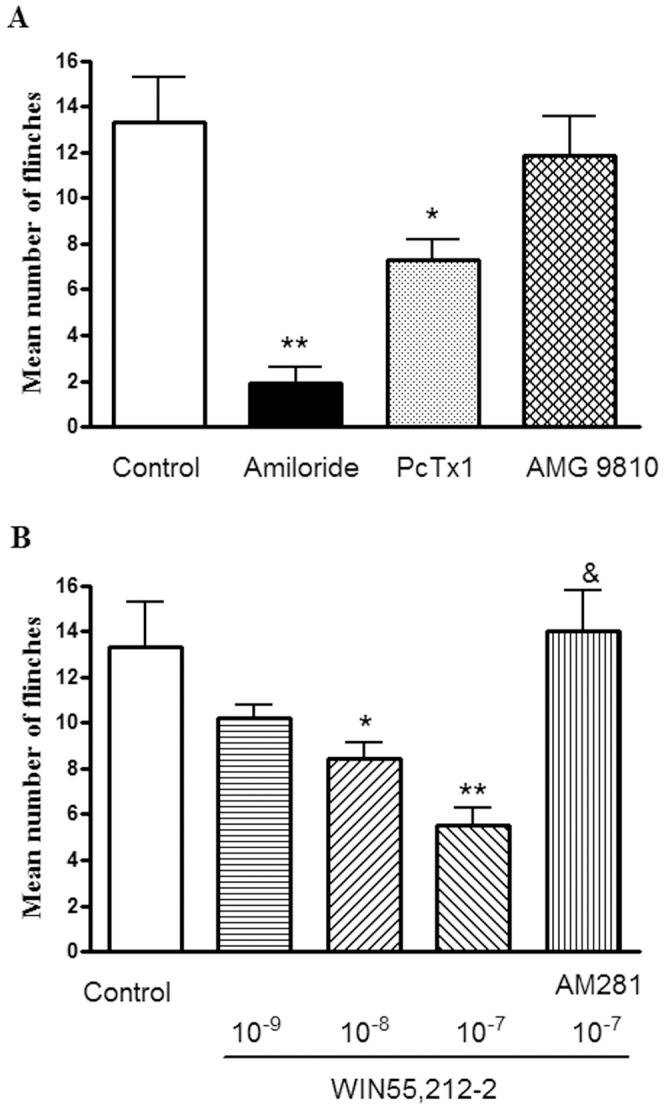
Effect of WIN55,212-2 on nociceptive responses to injection of acetic acid in rats. Intraplantar injection acetic acid (0.6%, 20 µl) evoked a flinch/shaking response. The bar graph in (A) shows acid-evoked pain was blocked by pretreatment of 200 µM amiloride, and partly blocked by pretreatment of 30 nM PcTx1. In contrast, the acid-evoked pain did not obviously change with pretreatment of 10 µM AMG 9810, *P<0.05, **P<0.01, unpaired t-test, compared with control. n = 8/each column. The bar graph in (B) shows the pretreatment of WIN55,212-2 (WIN) decreased flinching behavior induced by acetic acid in a dose-dependent manner. The effect of WIN55,212-2 was blocked by AM281 (10^−6^ M), a selective CB1 receptor antagonist. *P<0.05, **P<0.01, one way analysis of variance followed by *post hoc* Bonferroni’s test, compared with control; & P<0.01, *post hoc* Bonferroni’s test, compared with WIN (10^−7^ M) column. n = 8/each column. Flinching shaking of paw was recorded as the number of flinches per observation period (5 min).

Intraplantar injection of acetic acid elicited an intense flinch/shaking response in rats [11], [33]. The flinch response mainly occurred during 0–5 min after injection of acetic acid. Intraplantar injection of 20 µl acetic acid solution (0.6%) caused an intense flinch/shaking response (Fig. 7). And the acid-evoked pain was potently blocked by treatment of 200 µM amiloride, a broad-spectrum ASIC channel blocker. The number of flinches decreased from 13.3±2.0 of control conditions to 1.9±0.7 with 200 µM amiloride pretreatment (n = 8, P<0.01, unpaired t-test), suggesting the involvement of ASICs (Fig. 7A). Moreover, the acid-evoked pain was also partly blocked by 30 nM PcTx1, a specific antagonist of homomeric ASIC1a channels. The number of flinches decreased from 13.3±2.0 to 7.3±0.9 (n = 8, P<0.05, unpaired t-test). In contrast, the acid-evoked pain did not obviously change with pretreatment of 10 µM AMG 9810, an antagonist which can block proton activation of TRPV1. The number of flinches was 11.9±1.7 (n = 8, P>0. 1, unpaired t-test) (Fig. 7A).

The pre-treatment of WIN55,212-2 decreased flinching behavior induced by acetic acid in a dose-dependent manner (10^−9^ M–10^–7^ M) in eight rats/each group (Fig. 7B). The number of flinches decreased from 13.3±2.0 of control conditions to 5.5±0.8 with 10^−7^ M WIN55,212-2 pretreatment (n = 8, P<0.01, *post hoc* Bonferroni’s test). The effect of WIN55,212-2 on acetic acid-induced pain behaviour was blocked by AM281 (10^−6^ M), a selective CB1 receptor antagonist. The number of acid-evoked flinches was 14.3±1.8 with co-treatment of both 10^−6^ M AM281 and 10^−7^ M WIN55,212-2, and there was a significant difference compared with treatment of 10^−7^ M WIN55,212-2 alone (5.5±0.8, n = 8, P<0.05, *post hoc* Bonferroni’s test) (Fig. 7B). These results indicated that WIN55,212-2 relieved acidosis-evoked pain.

## Discussion

In this study we first report that CB receptor agonist WIN55,212-2 inhibited the activity of ASICs in the primary sensory neurons. WIN55,212-2 decreased the amplitude of ASIC currents, the number of action potentials induced by acid stimuli and nociceptive responses to injection of acetic acid. We further showed that CB1 receptor and cAMP-dependent signal pathway likely underlie intracellular mechanism in inhibiting ASIC activity by WIN55,212-2.

In agreement with previous studies, application of a pH 5.5 acid solution was found to evoke a slow-inactivating, rapid-inactivating or sustained inward current in native DRG neurons [34], [35]. All slow- and rapid-inactivating transient currents observed were ASIC currents, since they could be inhibited by the broad-spectrum ASIC channel blocker amiloride, not by the TRPV1 blocker AMG 9810. In contrast, sustained currents were partly blocked by amiloride or AMG 9810, suggesting ASIC and TRPV1 channels may be involved. All three types of acid-induced currents could be partly inhibited by 10 nM PcTx1, a specific antagonist of homomeric ASIC1a channels, suggesting ASIC1a subunit and other ASIC subunits mediated together these acid currents. An ASIC3-like current was revealed after the inhibition of the slow-inactivating transient current by application of PcTx1. Thus, proton activated mainly the members of the ASIC family rather than TRPV1. The present study shows that CB receptor agonist WIN55,212-2 had inhibitory effect on all three types of acid currents. Inhibition of ASIC currents by WIN55,212-2 cannot be due to the decrease in the affinity of ASICs to proton because this effect was observed at the saturating concentration of this agonist. WIN55,212-2 shifted the proton concentration–response curve downwards, with an decrease in the maximum current response but with no significant change in the EC_50_ and threshold values.

Unlike inhibiting effect of the CB receptor agonist WIN55,212-2 on ASIC currents, the CB-inactive enantiomer WIN 55, 212-3 failed to modulate proton-evoke currents, suggesting the involvement of CB receptor. In the present study, we concluded that WIN55,212-2 inhibitory effect was mediated by CB1 receptors, since the inhibition of proton-gated currents was blocked by the selective CB1 receptor antagonist AM 281, but not by the CB2 receptor antagonist AM630. It is been shown that CB1 receptors are abundantly expressed in the majority (70–80%) of nociceptive primary sensory neurons [16], [17], [18]. In contrast, CB2 receptors are expressed in a variety of immune cells and microglia. Activation of CB1 receptor leads to inhibition of AC and reduction of cAMP levels, which plays an important role in several aspects of cannabinoid function including modulating conductance of voltage-dependent K^+^ and Ca^2+^ channels [32], [36]. The inhibition of proton-gated currents by WIN55,212-2 may involve intracellular signal transduction, since AC activator forskolin and the addition of cAMP reversed the inhibition of WIN55,212-2. In cortical neurons, AKAP150 (a kinase-anchoring protein 150) has been shown to mediate a PKA-dependent phosphory of the ASICs, and inhibition of PKA binding to AKAPs reduces ASIC currents [37]. Thus, PKA may involve in the cAMP-dependence of inhibitory effect of WIN55,212-2 on ASIC currents.

ASICs are a family of cation channels and extracellar pH sensors. Activation of ASICs by proton can depolarize the neurons and generate action potentials [38]. ASICs are found to mediate cutaneous acid-induced pain [5], [39]. Intraplantar injection of acetic acid elicited an intense flinch/shaking response in rats, [11], [33]. The acid-evoked pain was significantly blocked by amiloride, a broad-spectrum ASIC channel blocker, indicating the involvement of ASICs. In the present study, behavioral experiments have demonstrated that WIN55,212-2 produced a cannabinoid-mediated analgesia in the acid infusion evoked pain via CB1 receptor. The results were also consistent with WIN55,212-2 inhibitory effects on acid–evoked action potentials in current clamp experiments. It has been shown that the activation of CB1 receptors by subcutaneous administration of URB937, a peripherally restricted inhibitor of fatty acid amide hydrolase, reduces visceral pain in the acetic acid model [40]. The antinociceptive effect of URB937 is blocked by the selective CB1 receptor antagonists AM251, but not by the CB2 receptor antagonist AM630 [40].

ASICs have been proposed to be involved in the perception of pain in conditions associated with tissue acidosis such as inflammation, ischemia, infection, tissue injury and tumor development [1], [2]. So ASICs have emerged as a potential therapeutic target for pain treatment. There is strong preclinical and clinical evidence already available supporting the role of CB1 receptor agonism in modulation of various pain states [41]. However, in humans the activation of CB1 receptors is associated with central adverse effects such as psychotropic effects, temporary memory impairment and dependence [42]. In this work we used the cell body of DRG neurons as a simple and accessible model to examine the characteristics of the membrane of peripheral terminals. It was been shown that CB1 receptors are synthesized in the bodies of primary sensory neurons and transported to their peripheral axonal branches [18], [43]. Histochemical analysis reveals the presence of ASIC2 and ASIC3 in cutaneous nerve endings [11], [44]. In the present study, our results strongly indicated cannabinoids can exert their analgesic action by interaction with ASICs in the primary afferent neurons.

In summary, we showed that the activity of ASICs can be inhibited by CB receptor agonist WIN55,212-2 in the primary sensory neurons. WIN55,212-2 decreased ASIC currents, action potentials and pain induced by acid stimuli. The inhibition was blocked by selective CB1 receptor antagonist, but not by CB2 receptor antagonist. Our results reveal a novel analgesic mechanism of cannabinoids by modulating native ASICs.

## Materials and Methods

### Isolation of the Dorsal Root Ganglion (DRG) Neurons

All experiments were approved by the Institutional Animal Care and Use Committee of Hubei University of Science and Technology (approval no.1094) and were carried out in strict accordance with the National Institutes of Health Guide for the Care and Use of Laboratory Animals. Two- to three-week old Sprague-Dawley male rats were anesthetized with ether and then decapitated. The DRGs were taken out and transferred immediately into Dulbecco’s modified Eagle’s medium (DMEM, Sigma) at pH 7.4. After the removal of the surrounding connective tissues, the DRGs were minced with fine spring scissors and the ganglion fragments were placed in a flask containing 5 ml of DMEM in which trypsin (type II-S, Sigma) 0.5 mg/ml, collagenase (type I-A, Sigma) 1.0 mg/ml and DNase (type IV, Sigma) 0.1 mg/ml had been dissolved, and incubated at 35°C in a shaking water bath for 25–30 min. Soybean trypsin inhibitor (type II-S, Sigma) 1.25 mg/ml was then added to stop trypsin digestion. After isolation, dissociated neurons were placed into a 35-mm Petri dish and incubated for >1 h in normal external solution before the start of electrophysiological experiments. The maximal incubated time was not more 5 h. The neurons selected for electrophysiological experiment were 15–35 µm in diameter.

### Electrophysiological Recordings

Whole-cell patch clamp and voltage-clamp recordings were carried out at room temperature (22–25°C) using a MultiClamp-700 B amplifier and Digidata-1440 A A/D converter (Axon Instruments, CA, USA). Recording pipettes were pulled using a Sutter P-97 puller (Sutter Instruments, CA, USA). The micropipettes were filled with internal solution containing (mM): KCl 140, MgCl_2_ 2.5, HEPES 10, EGTA 11 and Na_2_ATP 5; its pH was adjusted to 7.2 with KOH and osmolarity was adjusted to 310 mOsm/L with sucrose. Cells were bathed in an external solution containing (mM): NaCl 150, KCl 5, CaCl_2_ 2.5, MgCl_2_ 2, HEPES 10, d-glucose 10; its osmolarity was adjusted to 330 mOsm/L with sucrose and pH was adjusted to 7.4 with NaOH. DRG neurons were maintained in the external solution before they were used for electrophysiological experiments. The resistance of the recording pipette was in the range of 3–6 MΩ. A small patch of membrane underneath the tip of the pipette was aspirated to form a gigaseal and then a negative pressure was applied to rupture it, thus establishing a whole-cell configuration. The series resistance was compensated for by 70–80%. The adjustment of capacitance compensation was also done before recording the membrane currents. The membrane voltage was maintained at −60 mV in all voltage-clamp experiments unless otherwise specified. Current-clamp recordings were obtained by switching to current-clamp mode after a stable whole-cell configuration was formed in voltage-clamp mode. Only cells with a stable resting membrane potential (more negative than − 50 mV) were used in this study. Signals were sampled at 10 to 50 kHz and filtered at 2 to 10 kHz, and the data were stored in compatible PC computer for off-online analysis using the pCLAMP 10 acquisition software (Axon Instruments, CA, USA).

### Drug Application

Drugs used in the experiments were purchased from Sigma Chemical Co. and include hydrochloric acid, WIN55,212-2, WIN55,212-3,AM281, forskolin, 8-Br-cAMP, (E)-3-(4-t-butylphenyl)-N-(2,3-dihydrobenzo[b] [1], [4] dioxin-6-yl) acrylamide (AMG 9810), psalmotoxin 1 (PcTX1) and amiloride. Stocks of drugs were made up in dimethyl sulfoxide and diluted daily in the external solution at a minimum of 1∶1000 to a final working concentration. Next, they were hold in a linear array of fused silica tubes (o.d./i.d. = 500µm/200µm) connected to a series of independent reservoirs. The application pipette tips were positioned ∼30 µm away from the recorded neurons. The application of each drug was driven by gravity and controlled by the corresponding valve, and rapid solution exchange could be achieved within about 100 ms by shifting the tubes horizontally with a PC-controlled micromanipulator. Cells were constantly bathed in normal external solution flowing from one tube connected to a larger reservoir between drug applications.

### Nociceptive Behaviour Induced by Acetic Acid in Rats

Sprague-Dawley male rats (3–4 weeks) were kept with a 12-h light/dark cycle and with *ad libitum* access to food and water. Animals were placed in a 30 × 30 × 30 cm^3^ Plexiglas chamber and allowed to habituate for at least 30 min before nociceptive behaviour experiments. After the acclimation period, a blind experiment was carried out. Two intraplantar injections were made by using a 30-gauge needle connected to a 100-µl Hamilton syringe. The experimenters coded the animals and pretreated with amiloride, PcTx1, AMG 9810, WIN55,212-2 and/or AM281 in the dorsal face of the hind paw. After 5 min, the other experimenters subcutaneously administered 20 µl acetic acid solution (0.6%) into the same hind paw and observed nociceptive responses. The pH of the solution was 2.55 [33]. Nociceptive behaviour (i.e., number of flinches) was counted over a 5-min period starting immediately after the acetic acid injection [11], [33].

### Data Analysis

Data were statistically compared using the Student’s t-test or analysis of variance (ANOVA), followed by Bonferroni’s *post hoc* test. Statistical analysis of concentration–response data was performed using nonlinear curve-fitting program ALLFIT. Data are expressed as mean ± SEM.
